# Supplementary material for: Disparities in potential years of life lost due to intimate partner violence: Data from 16 states for 2006–2015
Source: PLoS One. 2021 Feb 17;16(2):e0246477. doi: 10.1371/journal.pone.0246477 (PMC7888591; doi:10.1371/journal.pone.0246477)
Supplement: Attachment — Submitted filename: PLOS ONE Article Review 10.07.20.docx [file pone.0246477.s001.docx]

**General Review of the Paper:**

The article “Disparities in potential years of life lost due to intimate partner violence: data from 16 states for 2006-2015” makes a significant contribution to the field of public health by exploring the societal costs of intimate partner violence (IPV). The literature review was well-written and sufficiently identified the gaps in existing IPV research. The focus on disparities as it related to both the number of IPV-related deaths and in the potential years of life lost due to IPV-related deaths is an innovative focus and significant contribution to the literature. The analyses also included analysis of perpetrator-victim relationships, which is important and informative for prevention programming. Limitations of the research included the use of a general life expectancy in order to address disparities, despite knowledge of disparities in life expectancy amongst racial groups; however, the authors offered a well-justified explanation for this decision. Additional feedback for the authors according to manuscript section is provided below.

**TITLE AND ABSTRACT**

- The title is appropriate.
- The abstract was comprehensive. One additional consideration might be the addition of the analyses documenting the victim -suspect relationship by fatality types.

**INTRODUCTION**

- This section summarizes the current literature and identifies the gaps in current research. It is well written and does a nice job justifying the research questions.
- Line 74-75: Are there any targeted prevention efforts that have been successful, or would these need to be developed and tested based on study results. Add a bit more clarification to this statement.

**METHODS**

- This section was well-written. There is one point that deserves more clarification (lines 121-125). Was a pre-existing NVDRS variable used or was a new variable created through narrative review (or a mixture of both).

**RESULTS**

- Clear and informative

**DISCUSSION**

- Note as a limitation that the differences in state density and racial and ethnic group composition among the 16 NVDRS states were not accounted for in analyses.
- Researchers should consider briefly mentioning other individual (SES, Education) and community level (crime, poverty level)variables that impact disparities that could be explored in future research.
- In reference to corollary victims being male more often than female, researchers might consider adding a statement about how young boys are subjected to physical child abuse more than girls.
  - A future study direction about exploring the ages of corollary victims could also be added.

**TABLES/FIGURES**

- The tables were generally informative and comprehensive.
- For Table 1, the inclusion of full-dataset number and percentages seemed unnecessary since the analysis was based on the reduced dataset.
