# Supplementary material for: Disparities in potential years of life lost due to intimate partner violence: Data from 16 states for 2006–2015
Source: PLoS One. 2021 Feb 17;16(2):e0246477. doi: 10.1371/journal.pone.0246477 (PMC7888591; doi:10.1371/journal.pone.0246477)
Supplement: Attachment — Submitted filename: Response to Reviewers.docx [file pone.0246477.s002.docx]

| **RESPONSE TO REVIEWERS**  The authors of this manuscript acknowledge and thank the reviewers for the time they spent reviewing this manuscript and offering thoughtful comments. We appreciated the positive comments made about the manuscript, and the critical comments helped us see ways in which we could strengthen our manuscript and research. We reviewed the recommendations and revised the manuscript in response the feedback provided by reviewers (see edits made in the manuscript in track changes). Additionally, we provide our detailed responses to reviewer comments in the table provided. | | | |
| --- | --- | --- | --- |
|  | **Editor/Reviewer Comments** | **Authors’ Responses** | **Changes to Manuscript** |
| **Editor** | | | |
| 1 | 1. Please ensure that your manuscript meets PLOS ONE's style requirements, including those for file naming. The PLOS ONE style templates can be found at  https://journals.plos.org/plosone/s/file?id=wjVg/PLOSOne_formatting_sample_main_body.pdf and  https://journals.plos.org/plosone/s/file?id=ba62/PLOSOne_formatting_sample_title_authors_affiliations.pdf | Thank you for this reminder. We have followed the *PLOS ONE* style requirements to the best of our ability. | See full manuscript |
| 2 | We note that you have indicated that data from this study are available upon request. PLOS only allows data to be available upon request if there are legal or ethical restrictions on sharing data publicly. For information on unacceptable data access restrictions, please see http://journals.plos.org/plosone/s/data-availability#loc-unacceptable-data-access-restrictions. | Thank you for this guidance. The data used in this study come from the National Violent Death Reporting System Restricted Access Data file (NVDRS RAD file). In order to access these data, we had to submit a proposal to the U.S. Centers for Disease Control and Prevention (CDC). The CDC reviewed the proposal and provided the RAD data once they approved the proposal, which include a Data Use Agreement signed by all parties with access to the data. Anyone who wishes to use these data must adhere to this same process, which is described here: <https://www.cdc.gov/violenceprevention/datasources/nvdrs/RAD.html>  The contact for inquiries about these data is: [nvdrs-rad@cdc.gov](mailto:nvdrs-rad@cdc.gov) | Because we cannot offer access to these data ourselves, we have described the legal reasons for this in our cover letter. |
| 3 | We note that you have provided funding information that is not currently declared in your Funding Statement. However, funding information should not appear in the Acknowledgments section or other areas of your manuscript. We will only publish funding information present in the Funding Statement section of the online submission form.  ii) Please remove any funding-related text from the manuscript and let us know how you would like to update your Funding Statement. Please include your amended statements within your cover letter; we will change the online submission form on your behalf. | We apologize for this error. We misunderstood where to place the information on funding support for the first author’s time and effort on this project as the funding was not awarded explicitly for work on this particular study. We have corrected this error. | We have deleted this information from our Acknowledgements and included instructions for how to change our Funding Statement in the cover letter for this re-submission. |
| **Reviewer 1** | | | |
| 4 | **INTRODUCTION**: Provide more statistics for the current incidence/trends of IPV-related deaths by race and sex. | Thank you for this helpful suggestion. We have included additional information on the only multistate U.S.-based study of which we are aware that has assessed rates of intimate partner homicide (IPH) and IPH-suicide for men and women by race/ethnicity. | Please refer to the additions to what is now the third paragraph of the Introduction. |
| 5 | **INTRODUCTION**: This paper does a good job explaining or providing footnotes on each category. Considering adding or clarifying if the suspect is included in the count for IPH-suicide. | Thank you for raising this question. Since this comment relates to the Introduction of the paper, we focused on adding clarification on this point to our description of background literature on the topics of IPH and IPH-suicide.  Just as a note, in terms of our study, deaths by suicide that were part of an IPH-suicide incident are included in the counts of intimate partner violence (IPV) fatality. The number of deaths by suicide can be seen in Table 2, partner victims, “self-inflicted” category for victim-suspect relationship. | Please see minor additions to the second paragraph of the Introduction and additional text in the third paragraph of the Introduction. |
| 6 | **METHODS**: Were any other covariates considered or limited by data sources? | We appreciate you raising this question. Given our research questions, we prioritized parsimonious models that focused on the intersection of race/ethnicity and sex, particularly given that this is the first study to examine potential years of life lost to IPV. However, we did explore the potential of including gender rather than sex in the analyses (i.e., information on transgender identities), but the data did not exist for most of the study period. Another variable of interest to our team given its potential importance was some measure of socioeconomic status; however, the data were not available to us. | We discuss limitations around including a more expansive gender variable in the “Strengths and limitations” subsection of the Discussion. We have also added more discussion in this subsection regarding additional individual-level and community-level indicators that should be explored in future research. |
| 7 | **METHODS**: Does this analysis account for regional differences? | Thank you for this question. If we understand the question correctly, we did not account for regional differences in the data, such as northeast, west, southeast, or other regions of the United States. Additionally, we find that the states included in these analyses do not fall neatly into particular regions of the United States. Given the methods for data collection and coding prior to data being submitted to the national data system (NVDRS), we found it important to account for potential clustering in the data at the state-level and did so using hierarchical linear modeling. | No changes made |
| 8 | **RESULTS** Table 2: Clarify in Table 2 if “Suspect was law enforcement” means that law enforcement caused the death. It is a little confusing in that there are potentially two suspects, the one who caused the death and the IPV perpetrator. | Thank you for this helpful suggestion. We have clarified this point in Table 2. | Please see updates made to Table 2 regarding the victim-suspect relationship category mentioned and table footnotes. |
| 9 | **DISCUSSION**: If appropriate, perhaps point out any known or informed conclusion that the impact of social distancing could have on IPV-related fatalities. | We appreciate you raising this point. As there is not a consensus conclusion on the potential impact of social distancing on IPV-related fatalities, we find ourselves unable to make a point about this issue. | No changes made |
| 10 | **DISCUSSION**: Consider including a comparison example of another disease or issues’ PYLL so the reader can have a general gauge on the total PYLL from IPV. | We appreciate the thoughtfulness of this suggestion. However, we find it inappropriate to directly compare PYLL from one disease with another disease or PYLL of violent deaths with another disease because PYLL depends on the average age at death and the commonality of the outcome (for example, average age at death from cardiovascular disease is much higher, so PYLL per death may be low, while average age for childhood cancers is very low, so the PYLL per death is very high). | No changes made |
| 11 | **DISCUSSION**: Discuss how generalizable these results are or aren't and how resource allocation and targeted IPV prevention strategies should also explore the paper’s results stratified by region. | These results are generalizable to the 16 US states the data come from. Given the data source (NVDRS), these deaths are not a sample of deaths; instead, these are all officially reported IPV-related fatalities in those 16 states from 2006-2015. As such, the PYLL figures reported in our study represent the actual lost societal potential in these states rather than an estimate. The confidence intervals simply show the uncertainty regarding the years someone may live. | We added additional language in the Conclusion to clarify that the results are generalizable only to the 16 states included in the data. |
| 12 | **DISCUSSION**: It might be worth bringing up some research on implicit bias toward IPV and likelihood of arrests/receiving support which could prevent fatalities. For instance if the victim is white, the suspect is more likely to be arrested. (McCormack PD, Hirschel D. Race and the Likelihood of Intimate Partner Violence Arrest and Dual Arrest. Race and Justice. September 2018. doi:10.1177/2153368718802352) | Thank you for raising this important and complex issue. We currently draw attention to the issue of systemic oppressions which might present barriers to receiving support in the fourth and fifth paragraphs of the Discussion. In response to this comment, we added a clarifying parenthetical statement to the fourth paragraph. We have respectfully opted not to add additional information on the specific topic of implicit bias as it might relate to arrests as a potential for fatality prevention because we find research in this area to be both underdeveloped and somewhat mixed. For example, see Koppa and Messing (2019). We found that we did not have ample space to dive further into this issue in a substantive way.  Koppa, V., & Messing, J. T. (2019). Can justice system interventions prevent intimate partner homicide? An analysis of rates of help seeking prior to fatality. *Journal of Interpersonal Violence*, 0886260519851179. | Please refer to the small addition made to the fourth paragraph of the Discussion. |
| **Reviewer 2** | | | |
| 13 | Limitations of the research included the use of a general life expectancy in order to address disparities, despite knowledge of disparities in life expectancy amongst racial groups; however, the authors offered a well-justified explanation for this decision. | Thank you for this feedback. | No changes made |
| 14 | **ABSTRACT**: One additional consideration might be the addition of the analyses documenting the victim -suspect relationship by fatality types. | Thank you for this suggestion. We have added a new sentence to the abstract related to this point. | Please see the new sentence in the “Methods” section of the abstract: “We describe fatalities by sex, race/ethnicity, age, and victim-suspect relationships…” |
| 15 | **INTRODUCTION**: Line 74-75 Are there any targeted prevention efforts that have been successful, or would these need to be developed and tested based on study results. Add a bit more clarification to this statement. | Thank you for highlighting this issue. Your feedback helped us further clarify our point. | Please see the expanded final sentence of the first paragraph of the Introduction. |
| 16 | **METHODS**: There is one point that deserves more clarification (lines 121-125). Was a pre-existing NVDRS variable used or was a new variable created through narrative review (or a mixture of both). | We appreciate you highlighting this area in need of clarification. We have used your feedback to slightly reorganize and expand the first paragraph of the “Sample selection” subsection of our Methods. | Please see the updated first paragraph of the “Sample selection” subsection of the Methods. |
| 17 | **DISCUSSION**: Note as a limitation that the differences in state density and racial and ethnic group composition among the 16 NVDRS states were not accounted for in analyses. | Thank you for this suggestion. We have added this point to our discussion of study limitations. | Please see the following addition to the “Strengths and limitations” subsection of the Discussion: “Additionally, we did not directly control for state-level population and racial and ethnic distributions as a fixed effect in our models; however, we controlled for state-level variation by considering state as a random effects variable.” |
| 18 | **DISCUSSION**: Researchers should consider briefly mentioning other individual (SES, Education) and community level (crime, poverty level) variables that impact disparities that could be explored in future research. | Thank you for this important suggestion. We have added more on this topic to the “Strengths and limitations” subsection of the Discussion in response your comment and comment 6. | Please refer to our additions to the “Strengths and limitations” subsection of the Discussion. |
| 19 | **DISCUSSION**: In reference to corollary victims being male more often than female, researchers might consider adding a statement about how young boys are subjected to physical child abuse more than girls. | Thank you for this suggestion. We respectfully have opted not to add this information to the manuscript because we find more research is needed to make this kind of connection between childhood abuse experiences and fatalities that result from IPV. | No changes made |
| 20 | **DISCUSSION**: A future study direction about exploring the ages of corollary victims could also be added. | We agree with your point and have added this to our Discussion. | We have added this point to the sixth paragraph of the Discussion. |
| 21 | For **Table 1**, the inclusion of full-dataset number and percentages seemed unnecessary since the analysis was based on the reduced dataset. | Thank you for this suggestion. Respectfully, we believe that showing this information for the full dataset increases the reader’s confidence in the study in that a reader can compare and see that the distribution of sex and age does not change between the full and reduced datasets. The age distributions are especially important for PYLL calculations. As such, we have opted to retain this information. | No changes made |
